# The orthodontic implant site-switching technique: a preliminary study in dogs

**DOI:** 10.1186/s13005-023-00373-2

**Published:** 2023-07-14

**Authors:** Meng Lu, Weixu Li, Yeqing Wang, Lixian Yuan, Meng Cao

**Affiliations:** Department of Orthodontics, School of Stomatology, The Fourth Military Medical University & State Key Laboratory of Military Stomatology & National Clinical Research Center for Oral Diseases & Shaanxi Clinical Research Center for Oral Diseases, Xi′an, 710032 China

**Keywords:** Orthodontics, Dental Implants, Micro-CT, Animal experiments

## Abstract

**Background:**

To evaluate the quantity and quality of bone in the newly formed edentulous area produced by the orthodontic implant site-switching technique.

**Methods:**

The bilateral maxillary first premolars of five beagle dogs were extracted and bone defects were created. The right and left sides of the maxilla were randomly divided into control and experimental sides. On the experimental side, the maxillary second premolar was mesially moved into the position of the missing first premolar. On the control side, the second maxillary premolar was extracted. Six months later, the beagles were euthanized. Microcomputer tomography was used to analyze bone microstructure parameters, alveolar bone height and alveolar bone width of the regenerated bone. Histological analysis was performed by staining tissue sections with toluidine blue.

**Results:**

Median BV/TV values in the experimental group (81.78%) were significantly larger than those in the control group (35.67%; p = 0.04). Median Tb.Sp values in the experimental group (0.14 mm) were significantly lower than those in the control group (0.54 mm; p = 0.04). Median Tb.Th values in the experimental group (0.48 mm) were significantly higher than those in the control group (0.21 mm; p = 0.04). Median Tb.Pf values in the experimental group (0.65/mm) were significantly lower than those in the control group (3.15/mm; p = 0.04). There was no significant difference in the trabecular number (Tb.N) between the two groups (p = 0.23). The median alveolar bone height values in the experimental group (-0.81 mm) were significantly higher than those in the control group (-2.11 mm; p = 0.04) at a distance 5 mm from the mesial CEJ of the third premolar. The median alveolar bone height values in the experimental group (0.45 mm) were significantly higher than those in the control group (-1.70 mm; p = 0.04) at a distance 6 mm from the mesial CEJ of the third premolar. There was no significant difference in alveolar bone width when compared between the two groups (p > 0.05).

**Conclusions:**

The newly formed edentulous area created by orthodontic treatment had more compact and thicker trabeculae than the extraction socket. Furthermore, the newly formed edentulous area had a greater alveolar bone height available for the placement of implants.

## Background

The long-lasting success of implant placement requires an adequate volume of hard tissue for circumscribing the whole implant for long term functional success [1]. Alveolar ridge resorption is a common side effect of tooth loss, trauma or infection and can significantly limit the placement of dental implants [2]. Numerous bone augmentation procedures have been shown to be effective, including guided bone regeneration, [3] distraction osteogenesis, [4] and onlay grafting [5]. However, the surgical augmentation of resorbed bone is associated with several limitations, including the reduced efficacy of vertical augmentation compared to horizontal augmentation and complications associated with the grafting technique [6]. Compared with invasive bone augmentation surgery, orthodontic-assisted implant therapy is a feasible and non-invasive method for the generation of new bone, particularly when patients require additional orthodontic therapy [7].

The application of orthodontic therapy in an attempt to enhance adjacent atrophic alveolar bone is referred to as the orthodontic implant site-switching technique (OISS) [6] and involves moving the adjacent teeth into the bone-deficient edentulous area, closing the edentulous space and creating an implant site adjacent to the original edentulous area [6, 8]. This technique has been linked to minor modifications in the periodontal supporting tissue of the shifting tooth. As the teeth migrate along the ridge, bone is deposited, thus restoring any dimensional defects [9]. Tooth movement towards the neighboring atrophic alveolar crest is accompanied by movement of the corresponding alveolar bone; this results in the enlargement of the alveolar ridge and eventually the elimination of the requirement for bone grafting [6].

However, very few research studies have investigated OISS; furthermore, most of the existing studies are case reports or series [10–15]. Consequently, there are two key issues that need to be addressed by dedicated research. First, following tooth extraction, the height and width of alveolar bone will decrease due to the lack of tooth support [16]. Because the alveolar bone in the newly established edentulous area is no longer adequately loaded after a single tooth moves to the atrophic alveolar ridge, it is possible that the size of the newly edentulous area will be similar to the atrophy of the alveolar ridge after an extraction. Secondly, although the newly established edentulous area arises from normal or near-normal tooth movement, the alveolar bone in this region is primarily an immature bundle of bone. Therefore, bone quality may not meet the specific requirements of implant placement. Based on this shortfall in information, the aim of the current experimental study was to evaluate the bone quantity and bone quality of the newly formed edentulous area when a tooth was moved to the atrophic alveolar ridge.

## Methods

### Subjects

Five healthy male beagle dogs (13 ± 0.6 months-of-age; mean weight: 12 ± 2 kg) were used in this study (Shaanxi Junxing Biotechnology Co., Ltd.). The experimental protocol was approved by the Institutional Animal Care and Use Review Committee of the School of Stomatology, Fourth Military Medical University (approval code: 2021kq-003). This study was performed in accordance with ARRIVE guidelines for preclinical animal studies. Adequate measures were taken to minimize pain or discomfort for the animals.

### Surgical procedure

The beagles were kept in individually cages at an ambient temperature of 25 °C and a relative humidity of 50 ± 10%, with a soft diet to minimize damage to orthodontic appliances. Researchers monitored beagles twice a day to determine their health status by monitoring their weight, food, and water intake. The dogs were fasted for 12 h prior to anesthesia. The dogs were sedated by an intramuscular injection of 0.1 ml/kg xylazine hydrochloride (Sumianxin, Fourth Military Medical University Laboratory, China). Anesthesia was induced with an intravenous bolus of 3–5 ml thiopental (Shanghai Xinya Co., Ltd.) followed by intubation and maintenance of anaesthesia by inhalation of 1.5% isoflurane (Rayward, China). 4 mg/kg Carprofen (Rimadyl®, Brazil) was injected subcutaneously into beagles for 5 days following invasive surgery to provide postoperative analgesia. Local anesthesia (2–4 ml lidocaine 2% with epinephrine 1:100 000) was used at the surgical site. All dogs were intramuscularly injected with penicillin (30,000 U/kg) three days after the operation and were fed liquid food for one week.

#### Defect creation

**Fig. 1:**
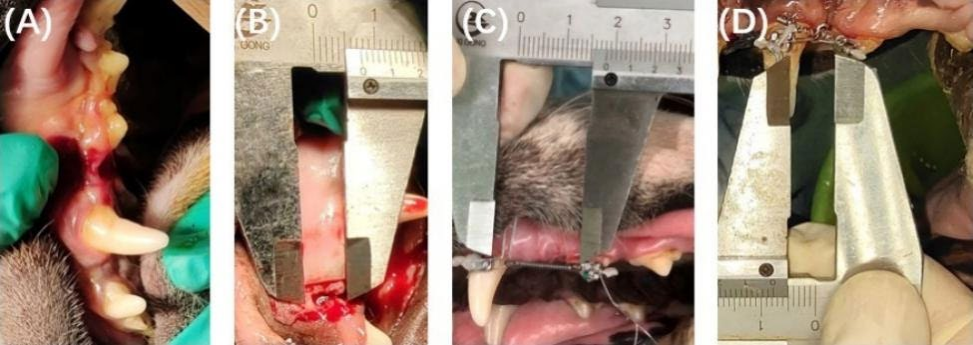
Intraoral images were taken at the main stages of the experiment. **(A)** Extraction of the maxillary first premolar; **(B)** The bone level was reduced in height in the area left by the extraction of the maxillary first premolars; **(C)** Orthodontic protraction of the second premolar into the defect using a canine as anchorage; **(D)** Final position of the second premolar

Prior to tooth extraction, 1% iodophor was used for oral disinfection. Bilateral first maxillary premolar extractions were performed on all dogs (Fig. 1A). Following a two-month healing period, the bone height in the area left by extraction of the first premolars was reduced. Full-thickness flaps were raised on the buccal side. Defects were created in the extraction region using diamond burs and saline irrigation. Bone defects (Fig. 1B) were created 2 mm from the second premolar’s mesial root and were approximately 5 mm in height (apico-coronally), 4 mm in depth (bucco-lingually) and 3 mm in width (mesio-distally). Then, the mucosa was adjusted and sutured. The healing of surgical defects took two months.

#### Tooth movement and tooth extraction

The experimental and control sides were randomly assigned to the right or left. The experimental side was chosen with the use of an electronically generated random number table.

In the experimental group, the maxillary second premolar was mesially moved into the position of the missing first premolar and formed a new edentulous area. The maxillary canine and the second premolar were etched with 37% phosphoric acid. Following the sealant was applied, brackets (0.022 in) were bonded to the buccal surfaces of the two teeth with Transbond X (3 M Unitek). A stainless-steel wire (0.018 × 0.025 in) was installed through the brackets of the second premolar and the canine. A nickel-titanium spring was then used to induce movement of the second premolar mesial with a force of 150 g. The appliances and oral hygiene were checked once a week, and the force magnitude was adjusted once a month (Fig. 1C, D). This will create a new edentulous area at the original second premolar of the experimental group.

At the same time, the maxillary second premolar was extracted in the control group. In theory, the alveolar bone condition following minimally invasive tooth extraction should be better than that following periodontal disease and even better than that following the traumatic loss of teeth. Therefore, this research aimed to compare the condition of alveolar bone in the newly formed edentulous area following orthodontic tooth movement with the best-case scenario of tooth loss. If the condition of alveolar bone in the newly formed edentulous area is better than the condition of alveolar bone after the healing of the extraction socket, it can be considered that the newly formed edentulous area can meet the requirements of implant placement.

### Retrieval of specimens

After six months of orthodontic treatment in the experimental group and six months of second premolar extraction healing time in the control group, all dogs were euthanized with an intravenous injection of an overdose of sodium pentobarbital (50 mg/kg). Maxillary specimens from the canine to the third premolar, were fixed in 10% formalin for one week.

### Micro-computed tomography (CT): reconstruction and analysis

**Fig. 2:**
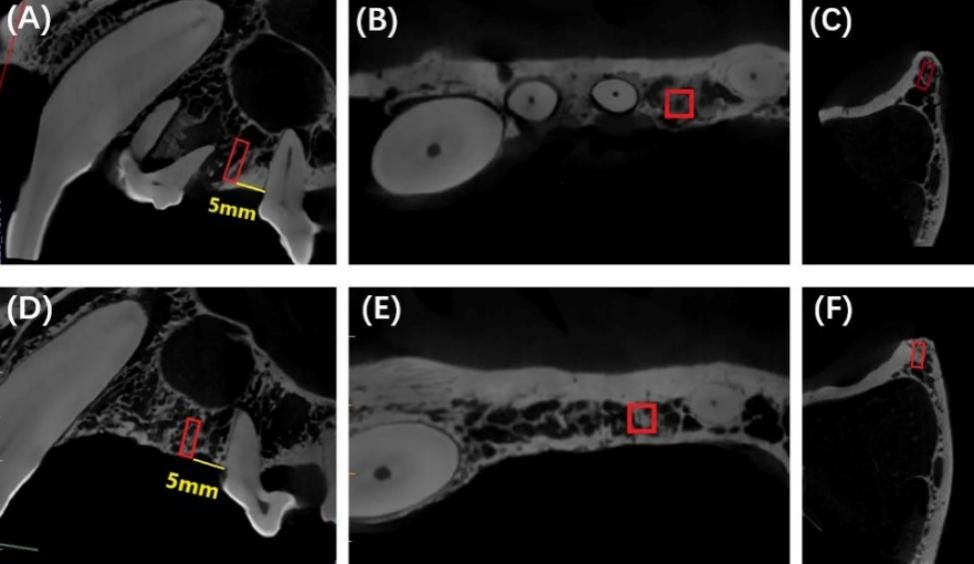
Regions of interest in the experimental group and the control group (red rectangular area). **(A)** Sagittal image of the experimental group; **(B)** Coronal image of the experimental group; **(C)** Axial image of the experimental group; **(D)** Sagittal image of the control group; **(E)** Coronal image of the control group; **(F)** Axial image of the control group

Micro-CT (Inveon, Siemens, Erlangen, Germany) was used to scan all samples at 30 μm resolution and with a source voltage of 80 kV and a current of 500 µA. Micro-CT software (Inveon Research Workplace) was used to reconstruct three-dimensional images for all specimens and analyze the key structural parameters of bone tissue. In both the experimental group and the control group, we selected a cuboid region of 1.5 mm (bucco-lingually) × 1.5 mm (mesio-distally) × 4 mm (apico-coronally) as the region of interest (ROI). And we selected an ROI that was 5 mm away from the mesial cementoenamel junction (CEJ) of the third premolar to ensure that both sets of ROIs were located in the same range of neogenesis on the left and right sides. The ROI in the experimental group was located on the alveolar bone in the new edentulous area, while the ROI in the control group was located on the tooth extraction socket (Fig. 2). The key measurement parameters included bone volume fraction (bone volume/total volume, BV/TV), trabecular thickness of new bone (Tb.N), trabecular number (Tb.N), trabecular separation (Tb.Sp) and trabecular bone pattern factor (Tb.Pf).

### The change of alveolar bone height of the second premolar

**Fig. 3:**
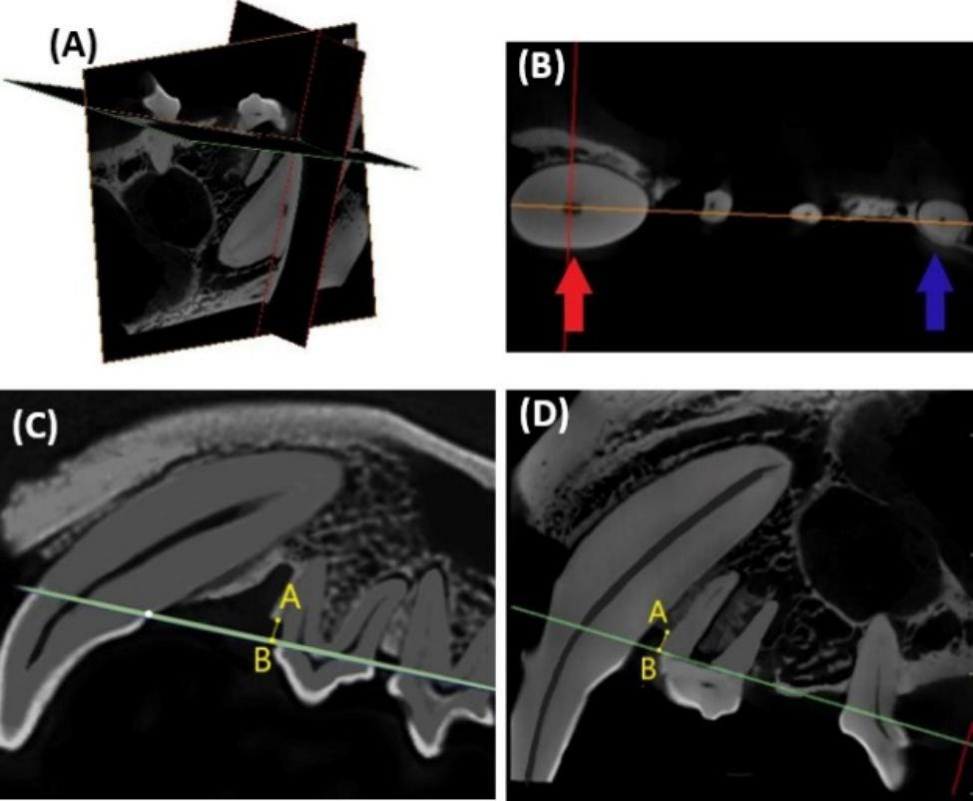
Measurement of the mesial alveolar bone height of the second premolar before and after orthodontic treatment. **(A)** Reference planes for the current position in the three-dimensional (3D) view; **(B)** The orange line through the center of the canine root (red arrow) and the center of the third premolar’s mesial root (blue arrow); **(C)** The mesial alveolar bone height (the distance between point A and point B) of the second premolar before orthodontic treatment. The green line was rotated through the canine’s distal cementoenamel junction (CEJ) and the third premolar’s mesial CEJ; **(D)** The mesial alveolar bone height (the distance between point A and point B) of the second premolar after orthodontic treatment

We measured the changes in the alveolar bone height of the second premolar before and after orthodontic treatment to see if periodontal support can be maintained when the second premolar is moved into a bony defect. Micro-CT scanning images were exported in DICOM format and imported into Mimics Research software (version 21.0; Materialise, Leuven, Belgium). As shown in Fig. 3A, a green line was drawn across the sagittal and axial images and used to indicate the exact location of the coronal image. The orange line indicates the exact location of the sagittal image. We reoriented all images with the canine and third premolar as benchmarks to ensure that the two groups of data were measured at the same positions before and after orthodontic treatment. The green line was rotated through the canine’s distal CEJ and the third premolar’s mesial CEJ (Fig. 3C, D), followed by the orange line through the center of the canine root and the center of the third premolar’s mesial root (Fig. 3B). This determined the placement of the three images. We took the vertical distance from the lowest point A of the mesial alveolar bone of the second premolar to the green line as the height of the alveolar bone. The greater the value, the more alveolar bone loss.

### Measurement of alveolar bone height in the new edentulous area

**Fig. 4:**
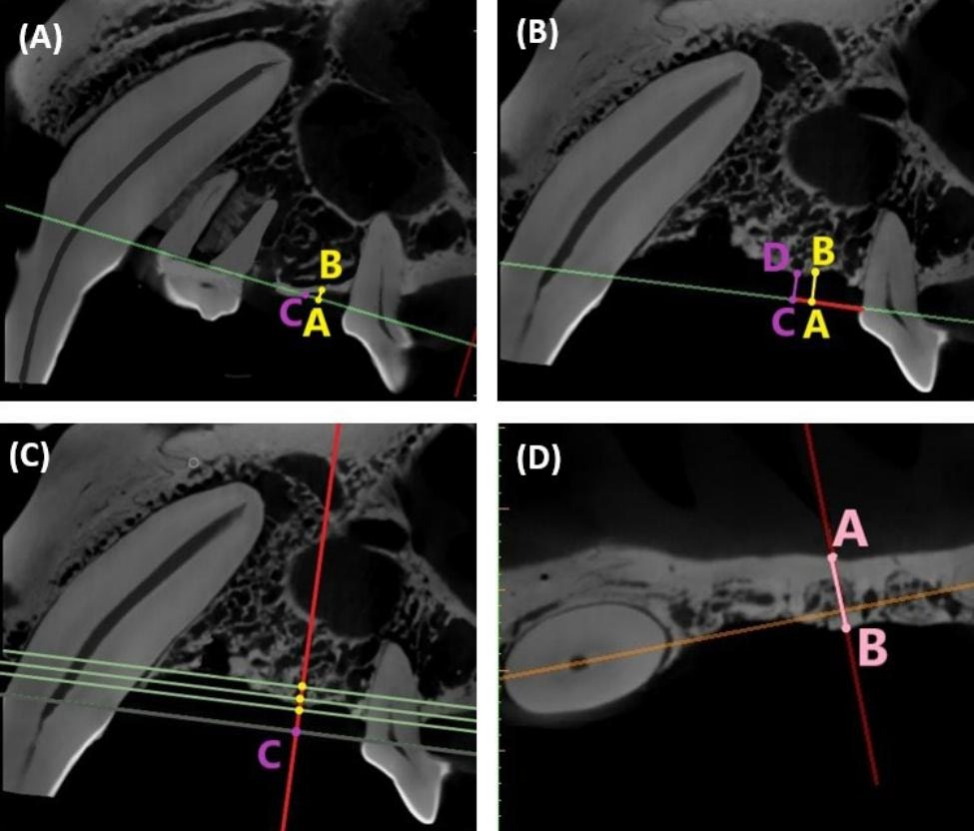
Measurement of alveolar bone height and width in the newly formed edentulous area. **(A)** Sagittal image from the experimental group. The green line was rotated through the canine’s distal cementoenamel junction (CEJ) and the third premolar’s mesial CEJ; **(B)** Sagittal image from the experimental group. The distance between point A and point B is the height of alveolar bone at a distance 5 mm from the mesial CEJ of the third premolar. The distance between point C and point D is the height of alveolar bone at a distance 6 mm from the mesial CEJ of the third premolar; **(C)** The green straight line was 2 mm, 3 and 4 mm away from point C; **(D)** Measurement of alveolar bone width on the coronal image (the distance between point A and point B)

We reoriented all images with the canine and third premolar as benchmarks in the same way as above. On the green straight line, a point A was selected that was 5 mm from the third premolar’s mesial CEJ. A straight line perpendicular to the green line was drawn through point A to contact the top of the alveolar ridge at point B. The distance between point A and point B represented the bone height (Fig. 4A, B). The same procedure was used to determine the height of the alveolar bone (the distance between point C and point D) at point C which was 6 mm from the third premolar’s mesial CEJ (Fig. 4A, B). The alveolar bone height was recorded as a positive value when point B was at the crown of the green straight line. The alveolar bone height was recorded as a negative value when point B was at the root of the green line. The greater the value, the greater the alveolar bone height.

### Measurement of alveolar bone width in the new edentulous area

Point C was determined as described above (Fig. 4C). The straight green lines were moved 2 mm, 3 and 4 mm parallel to the root of point C. The alveolar bone width (the distance between point A and point B) was then measured on three coronal images (Fig. 4D).

### Histological observations

The maxilla was segmented following micro-CT scanning. We retained the new edentulous area from the experimental group and the second premolar extraction socket from the control group for histological analysis. Specimens were immersed in a 10% buffered formalin solution for one day. Then, the samples were fixed in graded series of alcohols for three weeks, embedded in a solution of methacrylate and dibutyl phthalate, cut into 200 μm sagittal sections and ground to 90 μm slices using an Exakt Cutting/Grinding System (Exakt Apparatebau, Norderstedt, Germany). Toluidine blue was used to stain the sections which were then scanned by microscopy (Leica DM6000B Upright Research Microscope, Leica Microsystems, Wetzlar, Germany) to evaluate bone trabecular morphology.

### Statistical analysis

IBM SPSS Statistics for Windows, Version 26.0 (Armonk, NY: IBM Corp.) was used to analyze the data. The difference of bone tissue parameters, alveolar bone height and alveolar bone width between the two groups was analyzed using nonparametric Wilcoxon signed rank tests. For each variable and group, data were pooled per animal and the respective medians and quartile ranges were computed. The level of significance was set at p ≤ 0.05.

## Results

### Micro-CT morphometric analysis

**Fig. 5:**
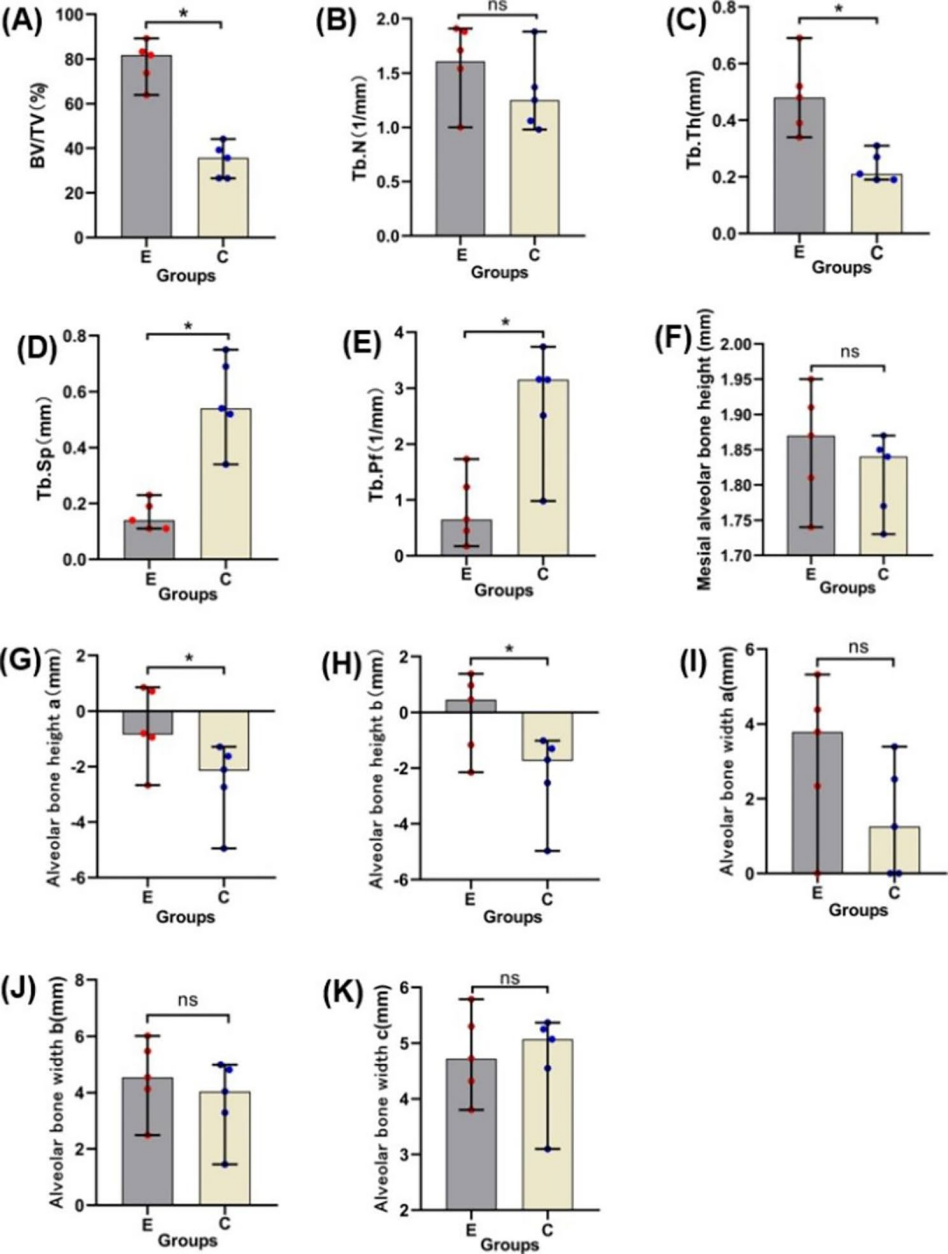
Micro-CT evaluation of the experimental groups and control groups. **(A)** Histomorphometric analysis of the percentage of bone volume (BV/TV), **(B)** Tb.N, **(C)** Tb.Th, **(D)** Tb.Sp and **(E)** Tb.Pf of ROIs in the regenerated bone area; **(F)** The change of mesial alveolar bone height of the second premolar before and after orthodontic treatment; (G) Alveolar bone height 5 mm from the mesial CEJ of the third premolar; **(H)** Alveolar bone height 6 mm from the mesial CEJ of the third premolar; **(I)** Alveolar bone width 2 mm from the root of point C; **(J)** Alveolar bone width 3 mm from the root of point C; **(K)** Alveolar bone width 4 mm from the root of point C (*p < 0.05; ns, no significant difference; E, experimental group; C, control group)

The BV/TV values in the experimental group (median: 81.78%; quartiles: 68.79 − 86.23%) were significantly larger than those in the control group (median: 35.67%; quartiles: 26.57 − 41.70%; p = 0.04; Fig. 5A). The Tb.Sp values in the experimental group (median: 0.14 mm; quartiles: 0.11 − 0.21 mm) were significantly lower than those in the control group (median: 0.54 mm; quartiles: 0.43 − 0.72 mm; p = 0.04; Fig. 5D). Tb.Th values in the experimental group (median: 0.48 mm; quartiles: 0.37 − 0.61 mm) were significantly higher than those in the control group (median: 0.21 mm; quartiles: 0.19 − 0.29 mm; p = 0.04; Fig. 5C). Tb.Pf values in the experimental group (median: 0.65/mm; quartiles: 0.31/mm − 1.48/mm) were significantly lower than those in the control group (median: 3.15/mm; quartiles: 1.75/mm − 3.45/mm; p = 0.04; Fig. 5E). There were no significant differences in Tb.N values when compared between the experimental and control groups (p = 0.23; Fig. 5B).

### The change of alveolar bone height of the second premolar

There was no significant difference in alveolar bone height of the second premolar before and after orthodontic treatment (p = 0.10; Fig. 5F). It showed that periodontal support was maintained when the second premolar was moved into the bony defect.

### Analysis of alveolar bone height in the new edentulous area

The height of alveolar bone in the experimental group (median: -0.81 mm; quartiles: -1.81 mm − 0.78 mm) was significantly higher than that in the control group [median: -2.11 mm; quartiles: -3.85 mm- (-1.46 mm); p = 0.04] at a distance 5 mm from the mesial CEJ of the third premolar (Fig. 5G). The height of alveolar bone in the experimental group (median: 0.45 mm; quartiles: -1.66 mm − 1.18 mm) was significantly higher than that in the control group [median: -1.70 mm; quartiles: -3.75 mm - (-1.16 mm); p = 0.04] at a distance 6 mm from the mesial CEJ of the third premolar (Fig. 5H).

### Analysis of alveolar bone width in the new edentulous area

There was no significant difference in alveolar bone width when compared between the experimental group and the control group (Fig. 5I, J, K).

### Histological findings

**Fig. 6:**
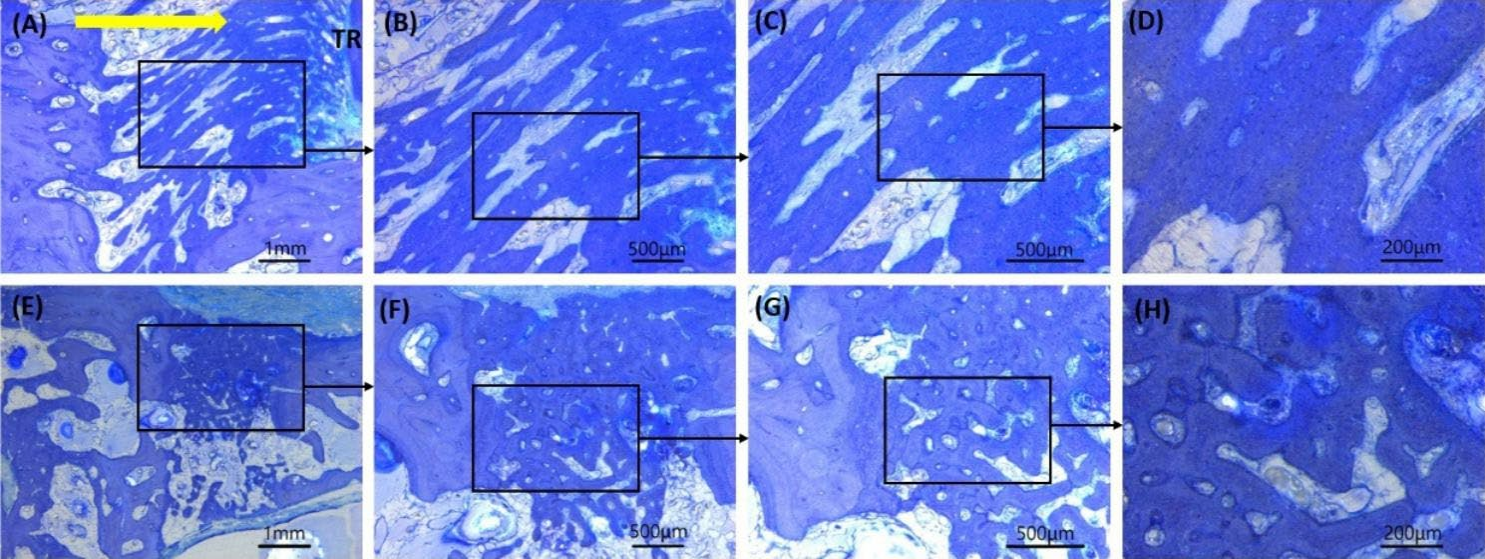
Histological analysis of the experimental group and control group. **(A)** New edentulous area in the experimental group; **(E)** Extraction socket of the second premolar in the control group; **(A)** and **(E)**, 20X magnification; **(B)** and **(F)**, 40X magnification; **(C)** and **(G)**, 60X magnification; **(D)** and **(H)**, 120X magnification; TR, root of the second premolar; Yellow arrow, the direction of tooth movement

The bone trabeculae in the new edentulous area produced by orthodontic treatment were thick, while the bone trabeculae in the extraction socket were short. In addition, the bone trabeculae in the new edentulous area were more closely combined (Fig. 6).

## Discussion

When a tooth is extracted due to periodontal disease or the tooth is congenitally missing, the alveolar ridge will be absorbed to varying degrees. Bone augmentation procedures need to be applied before the insertion of the dental implant for patients with bone deficiency. Compared with invasive bone augmentation surgery, OISS is a feasible and non-invasive method for the generation of new bone. The purpose of the current experimental study was to evaluate the bone quantity and bone quality of the newly formed edentulous area when a tooth was moved to the atrophic alveolar ridge.

To the best of our knowledge, this is the first study to evaluate the quantity and quality of the newly formed edentulous area when a tooth is moved to the atrophic alveolar ridge in a canine model. The anatomy of the first and second premolars, as well as the first and second molars, was comparable. When the bone quantity at the implant site is insufficient (due to a long period elapsing since the loss of teeth), an orthodontic procedure can be used to move another identical tooth to the area left by the missing tooth. The newly developed edentulous area may have sufficient bone for implant placement because it represents the consequence of natural tooth movement. In our study, we observed that periodontal support was maintained when the second premolar was moved into the bony defect. Besides, we observed that the newly formed edentulous area created by orthodontics exhibited more compact and thicker trabeculae when compared to the extraction socket. Furthermore, the newly formed edentulous area had more alveolar bone height available for implant placement.

A search of the existing literature identified only six articles relating to OISS. One of these six studies was a retrospective study, another was a case series, and the remaining four were case reports. Three of the case reports [10–12] described the successful use of OISS to produce implant sites with adequate bone quantity without performing bone grafting. However, one of these studies [13] reported issues with bone loss at the new implant site. This was most likely due to the immaturity of the bone at time of implant placement. Immature fascicular bone makes up the majority of the newly generated edentulous alveolar bone. Following orthodontic tooth movement, there may be a variation in ridge dimension loss in the newly developed edentulous region.

We used micro-CT to study bone quality in the new edentulous area in this study. BV/TV values represent the quantity of trabecular bone mass. An increase in this value shows that bone anabolism is greater than catabolism and that bone mass is increasing. We found that BV/TV values in the experimental group were significantly higher those in the control group, thus showing that the new bone formation in the new edentulous area generated by orthodontics was greater than that in the control group. The primary indicators for evaluating the structure of bone trabeculae are Tb.N, Tb.Th, and Tb.Sp. The mean number of trabeculae per unit length is expressed as Tb.N while Tb.Th denotes the thickness of the trabeculae and Tb.Sp refers to the distance between trabeculae. There were no significant differences in Tb.N values when compared between the experimental and control groups in our study. There was a significant difference in Tb.Sp and Tb.Th values when compared between the two groups, thus indicating that the bone trabeculae in the new edentulous area were more compact. Tb.Pf is a parameter that describes the proportion of lamellar structure and rod-shaped structure in the composition of bone trabecula; the higher the value, the more mature the trabecular bone. In our study, Tb.Pf values in the experimental group were significantly lower than those in the control group. These data showed that the bone trabeculae in the newly edentulous area were more mature than those in the tooth extraction area.

Two previous studies investigated dimensional alterations of the alveolar ridge after orthodontic tooth movement into edentulous regions of the alveolar ridge atrophy. One of these earlier studies [14] revealed that the newly constructed edentulous area had a reduced width; this reduction was most noticeable at a position 2 mm apical to the crest level (mean: 0.9 mm; range 0.5–1.2 mm) following orthodontic treatment [14]. At the one-year follow-up assessment, only slight variations in width were observed. According to another study, after 2–5 years of orthodontic treatment, the alveolar ridge width in the new edentulous area was reduced by 4.2% on average, and the alveolar ridge height decreased only marginally by 0.07 mm on average [15]. It should be noted, however, that these two previous studies used dental research models to evaluate dimensional changes in edentulous areas. This strategy may lead to measurement inaccuracies. Although orthodontic tooth movement is mostly made up of bodily movement with very small variations in three-dimensional directions, it cannot be ruled out that some unanticipated slight tooth movement may have affected the dimensional variations of the alveolar process. Consequently, these observations could imply that the increased width of the alveolar process resulted from the width of the teeth rather than from the development of new bone. These findings should also be viewed with caution. In our study, we found that at 5 and 6 mm from the mesial CEJ of the third premolar, the alveolar bone height in the experimental group was significantly higher than in the control group; there was no statistically significant difference in alveolar bone width when compared between the two groups.

This study had some limitations that need to be considered. One limitation is that the second premolar will invariably exhibit a slight inclination during the process of mesial movement. This is because the second premolar has two thick roots and moves a long distance. Another limitation is that our description of tooth movement does not fully represent the clinical situation. Thus, future studies should increase sample size and better control the overall movement of teeth.

## Conclusions

Our analysis showed that periodontal support was maintained when a tooth was moved into the bony defect. Besides, when compared to normal healing after tooth extraction, the bone quality of the new edentulous sites generated after the teeth had moved into the atrophic alveolar bone following orthodontic treatment was better at the same time point. Furthermore, the alveolar bone height in the new edentulous area was greater than that in the socket following tooth extraction. Thus, our data preliminarily confirm the feasibility of the OISS technique for clinical application.

## Data Availability

The data that support the findings of this study are available from the corresponding author upon reasonable request.

## List of abbreviations

OISS: orthodontic implant site-switching technique
Micro-CT: Micro-computed tomography
ROI: region of interest
CEJ: cementoenamel junction
BV/TV: bone volume/total volume
Tb.Th: trabecular thickness of new bone
Tb.N: trabecular number
Tb.Sp: trabecular separation
Tb.Pf: trabecular bone pattern factor
